# Whole-blood metabolic biomarkers improve detection of subclinical ketosis in postpartum grazing dairy cows

**DOI:** 10.3168/jdsc.2026-1117

**Published:** 2026-09-18

**Authors:** D.A. Vallejo-Timaran, N. Serna-Guerra, J. Chamorro-Arias, P. Betancourth-Chaves, F. Hernández-Oviedo, V.E. Castillo-Vanegas

**Affiliations:** 1 Corporación Colombiana de Investigación Agropecuaria–AGROSAVIA, Obonuco Research Center, 520038, Pasto, Colombia; 2 Agricultural Sciences Research Group–GIsCA, Faculty of Veterinary Medicine and Animal Sciences, Institución Universitaria Visión de las Américas, 050031, Medellín, Colombia; 3 Scientific Department, Veterinary Diagnosis Laboratory–VITALAB SAS, 054048, Rionegro, Colombia

## Abstract

Subclinical ketosis (SCK) is a common metabolic disorder during early lactation and is commonly identified using blood BHB concentration. Portable analytical systems allow metabolic biomarkers to be assessed under field conditions, but the diagnostic value of whole-blood glucose and lactate for SCK remains uncertain. This observational study with repeated measurements evaluated portable whole-blood biomarkers for identifying SCK in postpartum grazing dairy cows using BHB as the reference standard. A total of 259 observations from 96 clinically healthy postpartum cows managed under high-altitude grazing conditions were analyzed. Subclinical ketosis was defined as BHB ≥1.0 mmol/L. Serum glucose (SGLU), point-of-care blood analyzer glucose (EGLU), glucometer glucose (GGLU), and point-of-care blood analyzer lactate (ELAC) were evaluated. Inferential analyses accounted for within-cow dependence. The ELAC had the numerically highest area under the curve (0.693; 95% CI: 0.633–0.754), followed by EGLU (0.627; 95% CI: 0.557–0.697), GGLU (0.595; 95% CI: 0.516–0.675), and SGLU (0.482; 95% CI: 0.397–0.567). After Holm adjustment for pairwise receiver operating characteristic comparisons, only the difference between ELAC and SGLU remained significant. In cow-clustered logistic regression, higher ELAC was associated with greater odds of SCK (odds ratio = 1.74; 95% CI: 1.30–2.35), whereas lower EGLU was also associated with SCK (odds ratio = 1.04 per 1 mg/dL decrease; 95% CI: 1.01–1.07). Results were attenuated when SCK was defined using the more restrictive BHB threshold of ≥1.2 mmol/L. Lactate reflects a metabolic pathway distinct from ketone body accumulation and may provide complementary metabolic information; however, it should not replace direct BHB measurement for SCK identification. Further studies are needed to determine whether lactate adds diagnostic or prognostic value beyond BHB alone.

## Graphical Abstract

**Figure:**
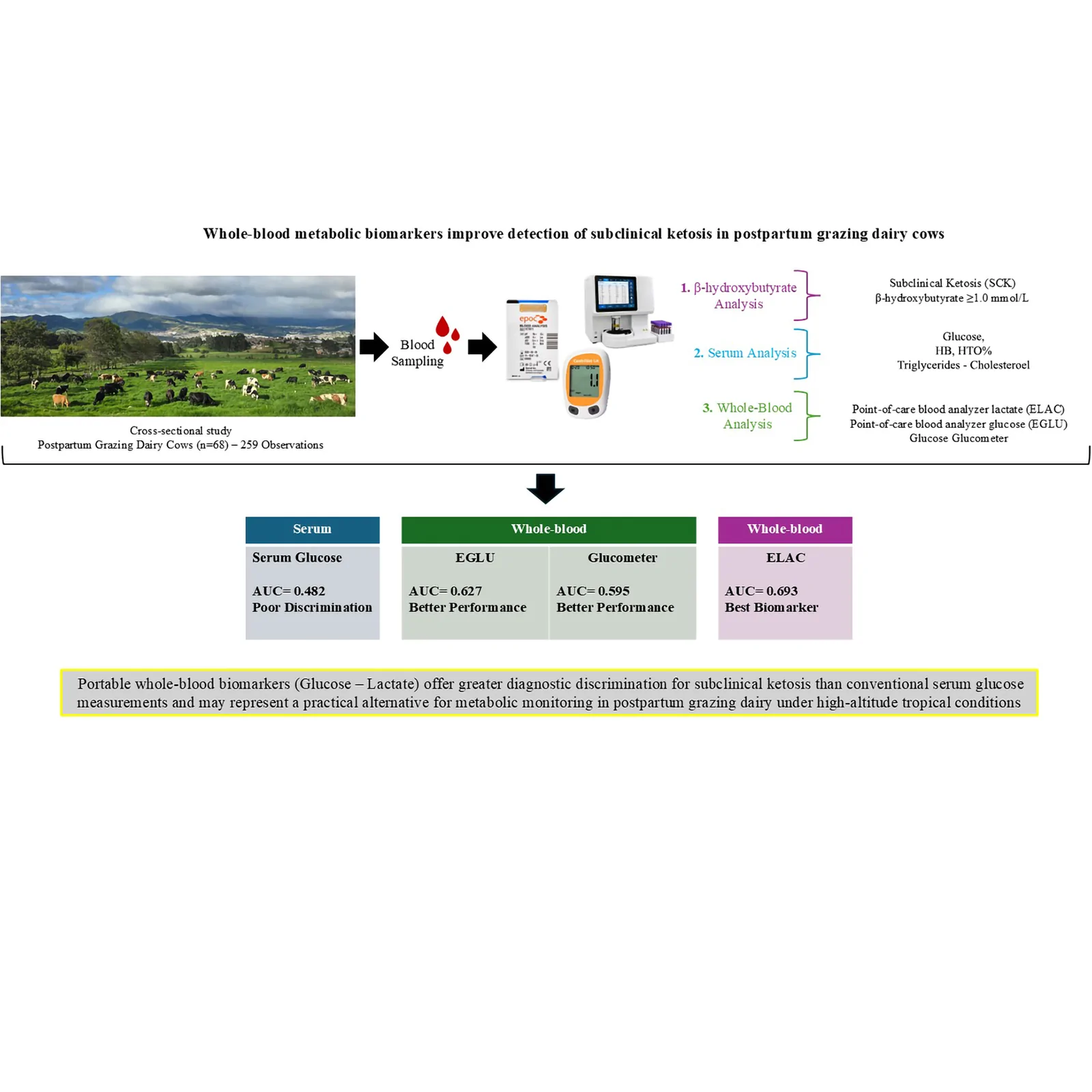

Subclinical ketosis (**SCK**) is one of the most common metabolic disorders affecting dairy cows during the transition period and early lactation. The disorder results from an imbalance between energy requirements and nutrient intake, leading to excessive mobilization of body reserves and increased hepatic ketogenesis. Elevated concentrations of BHB in the absence of clinical signs characterize SCK and have been associated with reduced milk production, impaired reproductive performance, increased risk of postpartum diseases, and economic losses (Raboisson et al., 2014). In grazing dairy systems, nutritional and environmental conditions exacerbate negative energy balance; thus, early identification of cows with metabolic disturbances remains an important challenge for herd health management (Vallejo-Timarán et al., 2021).

Measurement of BHB is widely accepted as the reference method for diagnosing SCK because circulating ketone bodies directly reflect alterations in energy metabolism (Rico and Barrientos-Blanco, 2024). However, blood or milk BHB testing may be limited under field conditions due to analytical costs, sample handling requirements, and limited access to laboratory facilities. Consequently, portable diagnostic technologies have gained interest as practical alternatives for on-farm metabolic monitoring. Whole-blood BHB and glucose (**GLU**) measurements obtained from handheld devices and point-of-care analyzers provide rapid results and facilitate real-time decision-making. Nevertheless, GLU concentration alone may not accurately reflect the severity of negative energy balance because GLU homeostasis is tightly regulated through endocrine and metabolic adaptations occurring during early lactation (Herdt, 2000; Rico and Barrientos-Blanco, 2024). Recent advances in portable analytical systems have expanded the range of measurable metabolites beyond GLU, enabling simultaneous assessment of multiple analytes from a single whole-blood sample. Among these, lactate has received increasing attention because of its central role in intermediary metabolism and its involvement in pathways associated with energy utilization and metabolic adaptation. Lactate concentrations may better reflect dynamic metabolic responses occurring during the transition period. Furthermore, integrated evaluation of GLU and lactate in whole blood may provide a more comprehensive assessment of metabolic status than isolated GLU measurements alone. Despite the growing availability of portable analyzers capable of measuring both metabolites, information regarding the diagnostic value of lactate for identifying SCK in grazing dairy cows remains scarce. We hypothesized that whole-blood measurements using portable analytical devices would provide greater diagnostic performance for detecting SCK than serum glucose (**SGLU**) concentrations. The objective of this study was to evaluate the diagnostic performance of portable GLU and lactate measurements for detecting SCK in postpartum grazing dairy cows using BHB as the reference standard.

An observational study with repeated measurements was conducted in grazing dairy cows under high-altitude conditions at the Obonuco Research Center, AGROSAVIA, Pasto, Colombia. The region is characterized by an altitude of 2,905 m, temperature of 12°C, and average precipitation of 1,100 mm. Cows were managed in a rotational grazing system based on fresh forage, with energy supplementation during lactation. The study population consisted of Holstein × Jersey cows during the first third of lactation.

Sample size was estimated as a priori for the agreement analysis between continuous diagnostic measurements. Assuming an expected concordance coefficient of 0.90 against a null hypothesis of 0.80, with α = 0.05 and 80% power, the sample size calculation was set at 60 paired measurements. The final analytical dataset included 259 measurements from 96 cows; statistical analyses accounted for within-cow dependence arising from repeated measurements. The number of observations per cow was variable (median of 2 observations, range 1–9). For cows with repeated measurements, consecutive sampling events were scheduled at 8-d intervals. Of the 96 cows, 38 (39.6%) contributed one observation, whereas 58 (60.4%) contributed repeated observations. Specifically, 15 (15.6%), 16 (16.7%), and 27 (28.1%) cows contributed with 2, 3, and ≥4 observations, respectively. Repeated measurements accounted for 221 of the 259 observations (85.3%). Clinically healthy cows without evidence of dystocia, retained placenta, systemic illness, or clinical reproductive disorders were included. Cows with clinical ketosis were excluded from the study population.

Blood samples were collected by coccygeal venipuncture before the morning milking. Serum GLU concentration was determined from blood collected in clot-activator tubes, centrifuged at 3,500 × *g* for 15 min at a controlled laboratory temperature of 18°C to 22°C, stored at −40°C, and analyzed using an enzymatic colorimetric GLU oxidase–peroxidase method. Whole-blood samples were collected in heparinized syringes and analyzed within 5 min of collection. Concentrations of GLU and BHB were determined using a portable meter (CentriVet GK, ANNAR Health Technologies, Mexico), whereas point-of-care blood analyzer glucose (**EGLU**) and point-of-care blood analyzer lactate (**ELAC**) were simultaneously measured using a point-of-care blood analyzer (EPOC Blood Analysis System, Siemens Healthineers, Germany). The SCK was defined according to the BHB concentration measured at each sampling event. Observations with BHB ≥1.0 mmol/L were classified as SCK, whereas observations with BHB <1.0 mmol/L were classified as non-SCK; therefore, repeated observations from the same cow refer to the classification of the cow at each sampling event rather than to a permanent classification of the animal across the study period. The lower threshold was selected in the present study to identify early increases in circulating BHB among clinically healthy postpartum cows, consistent with the preventive and early-detection objective of evaluating cow-side metabolic biomarkers. Because the choice of BHB threshold may influence diagnostic estimates, an additional sensitivity analysis was performed using the more restrictive and widely used cutoff of ≥1.2 mmol/L. In addition, hemoglobin (**HB**), hematocrit (**HTO**), cholesterol (**CHO**), and triglycerides (**TGC**) were measured to control metabolic factors that could influence association between energy metabolism and SCK.

Descriptive statistics were calculated for all variables. Comparisons of biomarkers between SCK and non-SCK cows were performed using *t*-tests; inferential associations with SCK were evaluated using regression models. Associations for GLU measurements were assessed using Pearson correlation. Because individual cows could contribute repeated measurements, 95% CI for correlation coefficients were obtained using cow-level bootstrap resampling clustered (**BRC**). Agreement between analytical methods was evaluated using Lin's concordance correlation coefficient and Bland–Altman procedures. The CI for concordance coefficients were obtained using cow-level BRC. For Bland–Altman analyses, repeated measurements were accounted for using mixed-effects models of the paired differences with a random intercept for cow, and 95% CI of agreement were estimated from the combined between- and within-cow variance. Diagnostic performance was assessed using receiver operating characteristic (**ROC**) curve analyses, with SCK classification based on BHB concentration as the reference standard. Area under the receiver operating characteristic curve (**AUC**) was estimated for ELAC and GLU measurements. The CI for AUC estimates and pairwise comparisons between ROC curves were obtained using cow level BRC. Univariable logistic regression models were fitted to estimate odds ratios (**OR**) and 95% CI for the association between each biomarker and SCK. For GLU measurements, OR were expressed per 1 mg/dL decrease in concentration, whereas the ELAC OR was expressed per 1 mmol/L increase. Within-cow dependence on logistic regression models was accounted for using cluster-robust SE at the cow level. As a sensitivity analysis, mixed-effects logistic regression models with a random intercept for cow were fitted. Additional sensitivity analysis was performed using BHB threshold of ≥1.2 mmol/L for SCK. To account for multiple testing, Holm's sequential adjustment was applied for the biomarker–SCK association tests and the pairwise comparisons of ROC curves. Statistical analyses were performed using Stata (Stata Corp. LLC, College Station, TX).

A total of 259 observations from 96 cows were included in the primary analysis. Mean GLU concentrations differ according to the analytical method and averaged 76.7 ± 15.5, 58.0 ± 11.0, and 68.6 ± 12.7 mg/dL for SGLU, EGLU, and glucometer glucose (**GGLU**), respectively. Mean BHB concentration was 0.99 ± 0.28 mmol/L. Based on a BHB threshold of ≥1.0 mmol/L, 136 of the 259 sampling records (52.5%) were classified as SCK. The SCK status was assigned independently to each sampling observation according to the corresponding BHB concentration. Mean ELAC concentration was 1.74 ± 1.15 mmol/L. Serum CHO and TGC averaged 102.0 ± 41.8 mg/dL and 56.9 ± 27.3 mg/dL, respectively. The HB and HTO values remained within physiological ranges. The SCK cows exhibited a metabolic profile characterized by higher BHB and ELAC concentrations with lower whole-blood GLU. The SGLU concentrations were similar between SCK and non-SCK cows (77.5 ± 15.7 vs. 78.0 ± 17.4 mg/dL). In contrast, whole-blood GLU concentrations were lower among SCK cows measured by EGLU (55.9 ± 10.6 vs. 60.2 ± 11.0 mg/dL) or GGLU (66.9 ± 12.6 vs. 70.5 ± 12.6 mg/dL). The ELAC concentrations were higher in SCK than non-SCK cows (2.05 ± 1.14 vs. 1.41 ± 1.08 mmol/L). The HB, HTO, CHO, and TGC concentrations showed limited variation and did not contribute to SCK discrimination.

Whole-blood EGLU and GGLU showed a strong positive correlation (r = 0.91; cow-clustered bootstrap 95% CI: 0.860–0.938), whereas correlations of SGLU with EGLU (r = −0.15; 95% CI: −0.261 to −0.009) and GGLU (r = −0.18; 95% CI: −0.333 to −0.042) were weak and negative. Concordance between EGLU and GGLU was moderate (Lin's concordance correlation coefficient = 0.645; cow-clustered bootstrap 95% CI: 0.558–0.714). Repeated-measures Bland–Altman analyses showed systematic differences among the analytical methods. The EGLU was, on average, 21.60 mg/dL lower than SGLU, with 95% limits of agreement (**LoA**) from −63.21 to 20.01 mg/dL, whereas GGLU was 10.53 mg/dL lower than SGLU, with 95% LoA from −55.18 to 34.12 mg/dL. Agreement was greater between the 2 whole-blood methods; however, EGLU remained systematically lower than GGLU, with a mean bias of −10.39 mg/dL and 95% LoA from −20.84 to 0.07 mg/dL (Figure 1).

**Figure 1:**
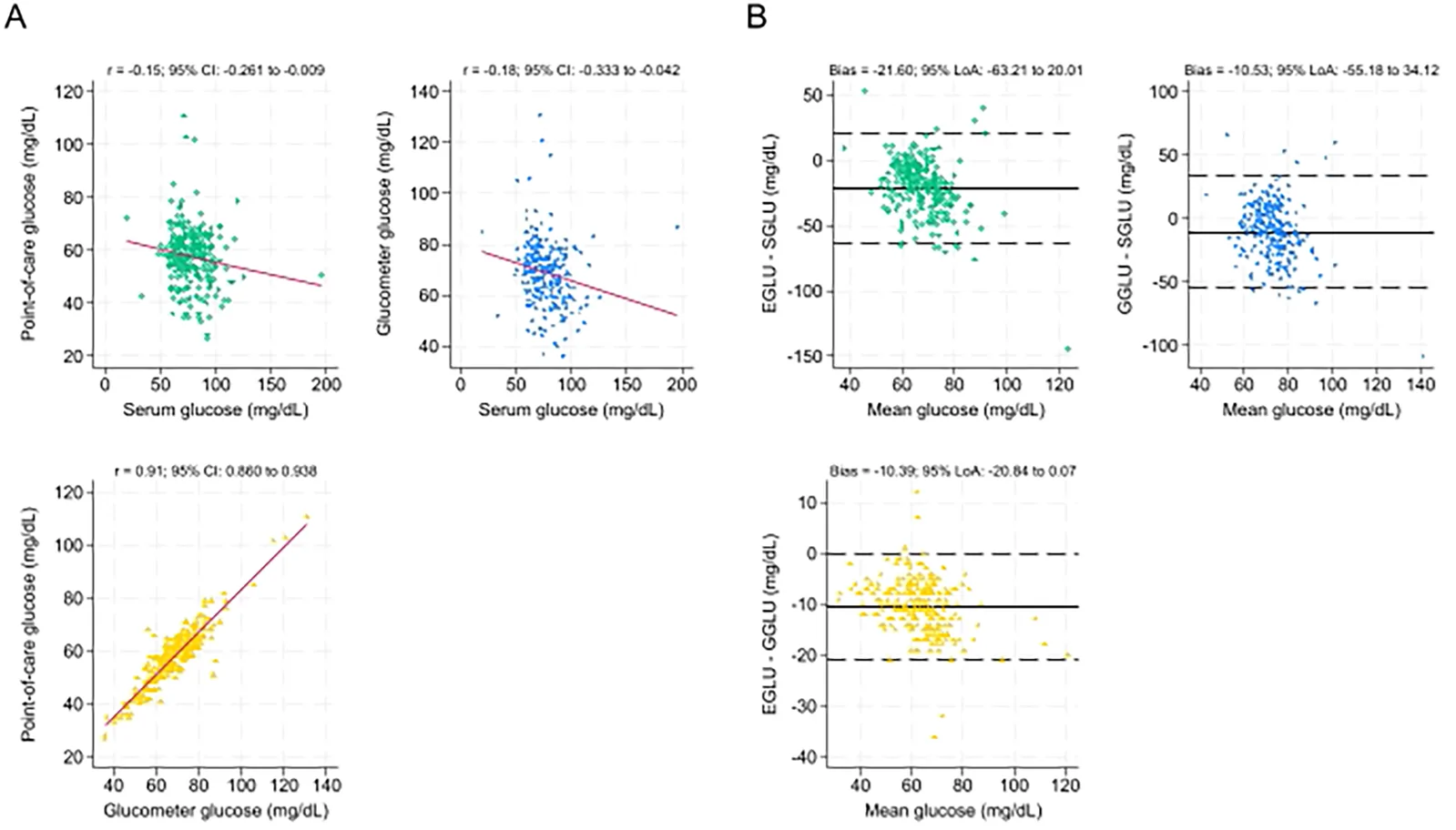
Association and agreement among serum and whole-blood glucose measurements in postpartum grazing dairy cows. (A) Pearson correlations for 3 pairwise comparisons: point-of-care blood analyzer glucose (EGLU) versus serum glucose (SGLU; upper left), glucometer glucose (GGLU) versus SGLU (upper right), and EGLU versus GGLU (lower left). Confidence intervals for correlation coefficients were obtained using bootstrap resampling clustered at the cow level. (B) Repeated-measures Bland–Altman analyses comparing the 3 analytical methods. Repeated measurements were accounted for using mixed-effects models of paired differences with a random intercept for cow. Solid horizontal lines represent the estimated mean bias, and dashed horizontal lines represent the 95% limits of agreement (LoA).

Associations between metabolic biomarkers and BHB concentration differed among analytical methods. β-Hydroxybutyrate was negatively correlated with EGLU (r = −0.193; cow-clustered bootstrap 95% CI: −0.303 to −0.070) and GGLU (r = −0.148; 95% CI: −0.256 to −0.024), whereas no association was observed with SGLU (r = −0.041; 95% CI: −0.156 to 0.080). Positive correlation was observed between BHB and ELAC (r = 0.209; 95% CI: 0.127–0.311). The HB, HTO, CHO, and TGC showed no associations with ketosis-related biomarkers. The ROC analyses showed that ELAC had the highest AUC (0.693; 95% CI: 0.633–0.754), followed by EGLU (AUC = 0.627; 95% CI: 0.557–0.697), GGLU (AUC = 0.595; 95% CI: 0.516–0.675), and SGLU (AUC = 0.482; 95% CI: 0.397–0.567). After Holm adjustment for 6 pairwise comparisons among ROC curves, the difference between ELAC and SGLU remained significant (adjusted *P* < 0.006). Thus, although ELAC showed the highest AUC point estimate, its discriminatory performance did not differ significantly from that of EGLU or GGLU after adjustment for multiple comparisons. In a sensitivity analysis using the more restrictive BHB threshold of ≥1.2 mmol/L, 53 of 259 observations (20.5%) were classified as SCK and showed minor associations and discriminatory performance compared with the ≥1.0 mmol/L threshold (Table 1, Figure 2). In cow-clustered univariable logistic regression analyses, each 1 mmol/L increase in ELAC was associated with greater odds of SCK (OR = 1.744; 95% CI: 1.296–2.346; *P* < 0.001; Holm-adjusted *P* < 0.004). Each 1 mg/dL decrease in EGLU was also associated with greater odds of SCK (OR = 1.040; 95% CI: 1.010–1.071; *P* = 0.009; Holm-adjusted *P* = 0.027). In contrast, GGLU (OR = 1.024 per 1 mg/dL decrease; 95% CI: 0.999–1.049; *P* = 0.061; Holm-adjusted *P* = 0.122) and SGLU (OR = 1.002 per 1 mg/dL decrease; 95% CI: 0.986–1.018; *P* = 0.827; Holm-adjusted *P* = 0.827) were not significantly associated with SCK. The present study showed that whole-blood metabolic biomarkers had higher numerical discriminatory performance than serum GLU for identifying SCK in postpartum grazing dairy cows. Among the evaluated biomarkers, lactate measured using a portable point-of-care analyzer had the highest AUC estimate, although its discriminatory performance differed significantly only from that of serum GLU after adjustment for multiple comparisons. Whole-blood GLU measurements also had higher AUC estimates than serum GLU; however, only EGLU remained significantly associated with SCK after Holm adjustment, whereas GGLU did not. These findings suggest that point-of-care measurement of whole-blood metabolic biomarkers may provide complementary information for on-farm metabolic assessment, whereas their diagnostic value for SCK requires further evaluation. This is particularly relevant given the high prevalence of SCK and its reported associations with reduced reproductive and productive performance (Duffield et al., 2009; Raboisson et al., 2014).

**Table 1:** Diagnostic performance and associations of metabolic biomarkers for the detection of subclinical ketosis in postpartum grazing dairy cows^1^

Variable	AUC (95% CI)	OR (95% CI)	*P*	Holm-adjusted *P*
SGLU (mg/dL)	0.482 (0.397–0.567)	1.002 (0.986–1.018)	0.827	0.827
EGLU (mg/dL)	0.627 (0.557–0.697)	1.040 (1.010–1.071)	0.009	0.027
GGLU (mg/dL)	0.595 (0.516–0.675)	1.024 (0.999–1.049)	0.061	0.122
ELAC (mmol/L)	0.693 (0.633–0.754)	1.744 (1.296–2.346)	<0.001	<0.004

1 AUC = area under the receiver operating characteristic curve; SGLU = serum glucose; EGLU = point-of-care blood analyzer glucose; GGLU = glucometer glucose; ELAC = point-of-care blood analyzer lactate; OR = odds ratio. Subclinical ketosis (SCK) was defined as BHB ≥1.0 mmol/L. The AUC estimates and 95% CI were obtained using bootstrap resampling clustered at the cow level and were based on 255 complete observations from 95 cows. Odds ratio and 95% CI were estimated through univariable logistic regression models with cluster-robust SE at the cow level and were based on available observations for each biomarker. For glucose measurements, the OR is expressed per 1 mg/dL decrease in concentration; for ELAC, the OR is expressed per 1 mmol/L increase. Holm-adjusted *P*-values account for multiple testing across the 4 primary biomarker–SCK association tests.

**Figure 2:**
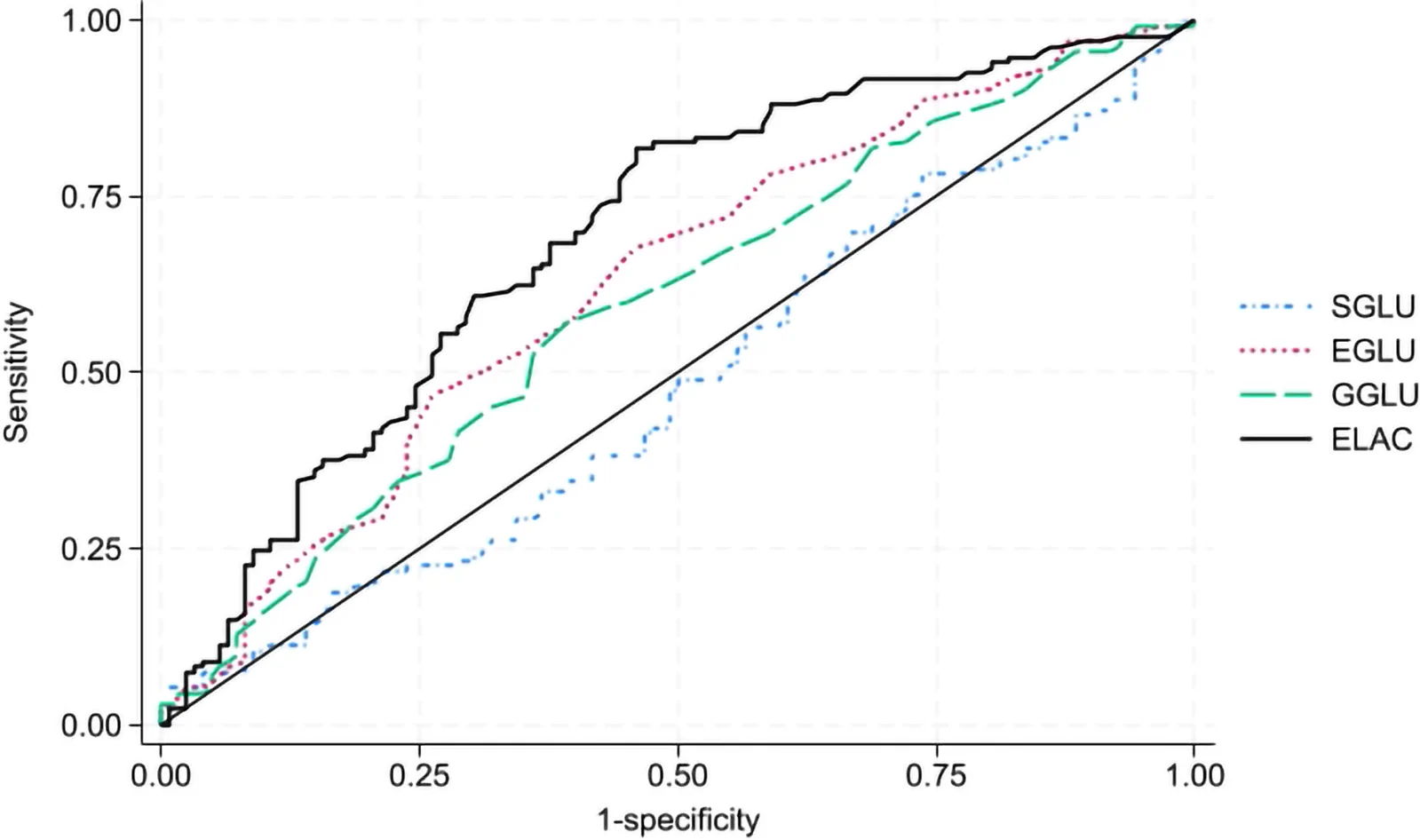
Diagnostic performance of serum and whole-blood metabolic biomarkers for detecting subclinical ketosis (SCK) in postpartum cows from a high-altitude grazing dairy system. Receiver operating characteristic (ROC) curves for point-of-care blood analyzer lactate (ELAC), point-of-care blood analyzer glucose (EGLU), glucometer glucose (GGLU), and serum glucose (SGLU), using SCK defined as BHB ≥1.0 mmol/L. Analyses included 255 complete observations from 95 cows. The ELAC showed the highest AUC (0.693; 95% CI: 0.633–0.754), followed by EGLU (0.627; 95% CI: 0.557–0.697), GGLU (0.595; 95% CI: 0.516–0.675), and SGLU (0.482; 95% CI: 0.397–0.567). After Holm adjustment for the 6 pairwise ROC comparisons, only the difference between ELAC and SGLU remained significant (adjusted *P* < 0.006).

The poor diagnostic performance observed for serum GLU was not unexpected. Negative energy balance during early lactation, when nutrient demands associated with milk synthesis exceed dietary energy intake, increase the risk of SCK (Baird, 1982; Herdt, 2000). Although this metabolic imbalance promotes adipose tissue mobilization and ketone body production, circulating GLU concentrations are maintained within physiological limits through endocrine and metabolic adaptations involving hepatic gluconeogenesis, insulin regulation, and peripheral nutrient partitioning (Herdt, 2000; Habel and Sundrum, 2020). Consequently, GLU concentrations fail to accurately reflect the severity of metabolic dysfunction, particularly in cows affected by subclinical disease (Oetzel, 2004). This physiological regulation explains the absence of differences in serum GLU between SCK and non-SCK cows and the lack of association between serum GLU and BHB concentrations. Thus, increased BHB may occur without a proportional decline in circulating GLU. The lack of a significant association between SGLU and SCK is consistent with previous reports showing that the relationship between GLU and BHB is variable in early-lactation dairy cows. Kauppinen (1983) reported a weak inverse correlation between BHB and GLU (r = −0.288), whereas Ruoff et al. (2017) showed that this relationship varied with parity and timing of ketosis. Blood GLU has shown limited diagnostic performance for identifying SCK (Asl et al., 2011).

In contrast, lactate emerged as an additional biomarker for identifying SCK because it serves as an important substrate for hepatic gluconeogenesis through the Cori cycle (Herdt, 2000). During periods of metabolic adaptation, increased lipid mobilization and altered carbohydrate metabolism may influence lactate production and utilization, generating metabolic signals that are not captured by GLU measurements alone. Recent advances in the understanding of ketone body biology suggest that metabolic adaptation during the transition period involves coordinated regulation of multiple substrates rather than isolated changes in GLU metabolism (Rico and Barrientos-Blanco, 2024). The positive association observed between ELAC and SCK suggests that lactate may reflect aspects of metabolic adaptation associated with increased ketone body production during negative energy balance. Although lactate has been investigated in relation to tissue perfusion, anaerobic metabolism, and illness, the present findings provide additional information regarding energy metabolism in transition dairy cows.

An additional finding of this study was the higher numerical discriminatory performance of whole-blood GLU measurements compared with serum GLU. The EGLU and GGLU were strongly correlated and showed higher AUC point estimates than SGLU, although these pairwise differences were not statistically significant after adjustment for multiple comparisons. These results may be partially explained by differences in sample matrix and preanalytical handling. Glucose concentrations in serum can be influenced by continued cellular glycolysis during sample storage and transport, resulting in progressive reductions in measurable GLU concentrations if sample processing is delayed. In contrast, portable analytical systems provide immediate measurements at the point of sampling and minimize the influence of preanalytical variation. Previous studies evaluating portable diagnostic technologies in dairy cattle have highlighted their value for rapid metabolic assessment and on-farm decision-making (Iwersen et al., 2009; Pineda and Cardoso, 2015). The correlation and agreement observed between the 2 whole-blood methods evaluated in the present study support the potential utility of portable technologies for field applications. However, the Bland–Altman results indicate that correlation and agreement should be interpreted separately. Both whole-blood methods showed negative biases relative to SGLU, and the wide LoA for EGLU versus SGLU (−63.21 to 20.01 mg/dL) and GGLU versus SGLU (−55.18 to 34.12 mg/dL) indicate substantial disagreement at the individual measurement level. Therefore, the correlation between the 2 whole-blood methods should not be interpreted as evidence of direct interchangeability. Our results indicate that GLU concentrations should be interpreted according to the diagnostic method used, particularly when serial measurements are used for metabolic monitoring.

The practical implications of these findings are particularly important for grazing dairy systems. In many pasture-based production systems, access to laboratory facilities may be limited, increasing dependence on diagnostic tools that can be implemented directly at the farm level. Previous studies conducted under grazing conditions have reported substantial prevalence of SCK and emphasized the importance of early identification of affected animals (Vallejo-Timarán et al., 2020, 2021). Under these conditions, portable whole-blood analyzers may facilitate real-time metabolic monitoring and support management decisions aimed at reducing the consequences of transition disorders. From a clinical perspective, ELAC should not be considered a replacement for direct BHB measurement. β-Hydroxybutyrate remains the reference biomarker for identifying SCK and directly reflects ketone body accumulation. In contrast, lactate represents a different metabolic pathway related to pyruvate metabolism, cellular redox balance, and the interconversion of lactate and pyruvate, and may therefore provide information on metabolic adaptations that is not captured by BHB alone. Thus, the potential value of ELAC lies not in replacing BHB, but in providing complementary metabolic information as part of a multiparametric point-of-care assessment. However, the moderate discriminatory ability observed for ELAC in the present study indicates that lactate alone is insufficient for diagnosing SCK. Further research is needed to determine the biological basis and clinical relevance of the association between lactate and SCK, particularly whether incorporating lactate into multiparametric approaches provides additional diagnostic or prognostic information beyond direct BHB measurement. The HB, HTO, CHO, and TGC did not contribute to the discrimination of SCK in the present study. Although these variables may reflect broader aspects of physiological status and lipid metabolism, their individual diagnostic performance was limited. These findings suggest that biomarkers associated with energy metabolism and ketogenesis may be more informative for identifying cows experiencing metabolic adaptation during early lactation. Blood BHB cutoffs used to identify SCK varied across studies, with values ranging from 1.0 to 1.4 mmol/L, although ≥1.2 mmol/L is the most frequently used threshold (Benedet et al., 2019). The ≥1.0 mmol/L threshold used in the present study was selected to detect early increases in circulating BHB in clinically healthy postpartum cows. Previous studies have shown that increases in BHB within this range are associated with adverse health and productive outcomes (Duffield et al., 2009; Ospina et al., 2010).

Several limitations should be considered when interpreting these results. The study was conducted in a single herd of grazing dairy cows under high-altitude tropical conditions; consequently, the external validity of the findings may be limited, and extrapolation to other herds, breeds, management conditions, or production systems should be undertaken with caution. Although repeated measurements were available for most cows and within-cow dependence was accounted for in the statistical analyses, the observational design and variable number of measurements per cow limited assessment of temporal relationships between biomarker changes and subsequent metabolic outcomes. Diagnostic estimates were also sensitive to the BHB threshold used to define SCK. When the more restrictive threshold of ≥1.2 mmol/L was applied, the proportion of observations classified as SCK decreased from 52.5% to 20.5%, reducing biomarker associations and discriminatory performance. The findings at the ≥1.2 mmol/L threshold should not be interpreted solely as evidence of poorer biomarker performance, and further studies involving larger numbers of independent animals with different BHB threshold across multiple herds and production systems are necessary to validate these findings.
